# Whipple’s disease: a fatal mimic

**DOI:** 10.4322/acr.2020.237

**Published:** 2021-01-28

**Authors:** Benjamin Kukull, Jonathon Mahlow, Gillian Hale, Lindsey J. Perry

**Affiliations:** 1 University of Utah School of Medicine, Department of Pathology, Salt Lake City, UT, USA

**Keywords:** Autopsy, Communicable Diseases, Pathology, Polymerase Chain Reaction, Tropheryma

## Abstract

Whipple’s Disease, a rare diagnosis caused by the slow-growing bacterium *Tropheryma whipplei*, most often presents with the classically described signs of malabsorption due to gastrointestinal colonization. However, it can also have signs and symptoms that clinically overlap with rheumatic diseases, potentially resulting in misdiagnosis. Furthermore, treatment with modern potent biologic immunosuppressive agents and classic disease modifying anti-rheumatic drugs (DMARDs) can lead to serious exacerbation of undiagnosed infections. We present the case of a middle-aged woman with long term complaints of arthalgias, who was diagnosed with seronegative rheumatoid arthritis and subsequently treated for almost 7 years with such immunosuppressive therapies. The patient’s disease course included chronic diarrhea that abruptly intensified and culminated in fatal hypovolemic shock/sepsis. A diagnosis of WD was made by autopsy examination, wherein several organ systems were found to be heavily involved by *Tropheryma whipplei* organisms, and their identification was confirmed with histochemical and molecular evaluation. Notably, most bacterial organisms were located deeply in the submucosa/muscularis of affected organs, a practical reminder to practicing pathologists that challenges the classic histopathologic description of Whipple disease as an infiltration of predominantly lamina propria, and the potential for sampling bias in typically superficial endoscopic biopsies during routine procedures.

## INTRODUCTION

Whipple’s disease (WD) is an exceedingly rare systemic infection caused by the bacterium *Tropheryma whipplei* (*T. whipplei*), a fastidious, bacilliform, phylogenetically gram-positive bacteria characteristically positive for periodic acid schiff histochemical stain and diastase resistant (PAS-D+).^1^ WD presents with non-specific gastrointestinal symptoms and malabsorption. The disease reportedly predominantly affects middle-aged/elderly men (the majority of reported cases are in men over 55 years old), which is purportedly due to its association with wastewater workers and farmers (who are predominantly male) and the smoldering disease course that can take many years to manifest and correctly diagnose.^2^ ^-^ ^4^ However, recent epidemiological studies have suggested that the contemporary prevalence of WD may be comparable in men and women in the US.^5^

The manifestations of WD are non-specific and may mimic other chronic disseminated infections as well as autoimmune disease. Symptoms of WD include, diarrhea, abdominal pain, adenopathy, arthralgias, and wasting with long-standing untreated disease. A prodromal stage has been reported with arthralgias years before the manifestation of gastrointestinal symptoms/malabsorption.^6^ ^-^ ^8^ The features of chronic arthralgias of Whipple’s disease overlap with the more prevalent degenerative and rheumatologic arthropathies,^8^ which - especially in women – can challenge efforts at diagnosis. We also note that the rheumatologic arthropathies can be concomitant with WD, which may further prolong the path to the correct diagnosis.

Due to its rarity, a diagnostic work-up for WD generally takes place after more common disorders with overlapping presentations are ruled out. In these cases, WD is classically diagnosed with tissue biopsy of the small intestine or enlarged lymph node followed by PAS-D stain to visualize *T. whipplei* intracellularly in macrophages. PAS staining is not sensitive or specific for *T. whipplei*, so additional molecular tests are usually performed to confirm the diagnosis. These tests may include *T. whipplei*-specific immunohistochemical (IHC) stains or detection of amplicons by polymerase chain reaction (PCR) directly from tissue sections or body fluids.^3^ ^,^ ^9^ ^,^ ^10^

Several organ systems can concurrently be involved by WD, which may account for its diversity and severity in presentation. Progressive central nervous system involvement is among the most serious complications, and is most commonly seen as a triad of dementia, supranuclear ophthalmoplegia and myoclonus.^11^ Cardiac involvement presents with murmurs and eventual heart failure due to valvular colonization and degeneration, which may necessitate valve replacement.^12^ ^,^ ^13^ Treatment consists of long term antibiotic regimens and is mostly effective.^1^ However, left untreated, WD is thought to have a ubiquitously poor prognosis with long term malabsorption leading to severe cachexia, and eventually death.

Herein, we present a case of WD mimicking seronegative rheumatoid arthritis in a middle-aged female treated with long term immunosuppressive therapy, culminating in severe diarrhea, hypovolemic shock and death.

## CASE REPORT

A 58-year-old female presented to the emergency department (ED) as a transfer patient from another institution with undifferentiated shock, right bundle branch block, and a history of chronic diffuse, watery, non-bloody diarrhea that markedly worsened for one day duration. The patient had just recently moved from another state; her outside medical records were unable to be obtained making the true chronicity of some of her symptoms challenging to determine - it appears most were present for approximately 7 years in a relapsing-remitting fashion. Initial vitals on admission to the ED included blood pressure of 60/40 mmHg, a heart rate of 116 BPM, and a temperature of 35.9 C. No cardiac valvular dysfunction was noted during the physical examination at this time. Her clinical history included years of persistent fever of unknown origin, migratory arthralgias, nausea, and nonspecific neurologic symptoms (headache, unilateral visual impairment, and paresthesia of left upper extremity). Due to this clinical picture, a working diagnosis of seronegative rheumatoid arthritis (RA) was proposed several years prior. Her general symptoms had persisted and worsened since this time despite almost 7 years of therapy with a number of immunosuppressive and disease-modifying anti-rheumatic drugs (DMARDs), which included: tocilizumab (an interleukin-6 receptor antagonist), leflunomide (a pyrimidine synthesis inhibitor), anakinra (an interleukin 1 receptor antagonist), rituximab (an anti-CD20 antibody, reducing B cell responses), hydroxychloroquine, and prednisone.

Several imaging studies were performed to locate the cause of the patient’s hypovolemia. X-ray and computed tomography imaging showed probable chronic inflammatory changes of the cecum and ascending colon, mild edema throughout the mesentery and fluid in the peritoneum and paracolic gutters, several calcified mesenteric lymph nodes, pleural thickening and effusion. A PET scan showed hypermetabolic activity of the lower esophagus to the cardia and of several of the partially calcified lymph nodes and diffuse hepatic steatosis. No sources of internal hemorrhage were noted.

At the ED, attempts were made to correct severe hypotension and metabolic disturbances, but they were refractory to both pressors and IV fluids. Because the etiology of her hypovolemic shock was unknown, she also received broad spectrum antibiotics (including vancomycin and piperacillin/tazobactam), and steroids in an attempt to broadly cover bacterial sepsis. Her various laboratory studies after admission included: a metabolic (lactic) acidosis reaching a low of pH 6.8, mild leukocytosis (13.3 k/uL [ reference range (RR); 4.30-11.30 k/uL]), critically high potassium (7.5 mmol/L [RR; 3.5-5 mmol/L]), low total protein (3.8 g/dL [RR; 6.5-8.5 g/dL]), critically low hemoglobin (5.9 g/dl [RR; 12.6-15.9 g/dL]), severe coagulopathy (Pt Time: 42.2 sec [RR;12-15.5 sec]; platelets 31 k/uL [RR; 159-439 k/uL]), signs of multi-organ damage, and eventual complete liver failure (last measured AST = 2792 U/L [RR; 16-40 U/L],ALT = 1625 U/L [RR; 5-60 U/L], albumin (1.9 g/dL [RR; 3.5-5.0 g/dL]), and total bilirubin 1.4 mg/dL (0.2-1.4 mg/dL). Blood cultures did not yield any growth (even after her eventual demise). Despite multiple successful resuscitation attempts during the course of her hospitalization, her critical condition was refractory to treatment, and she was declared dead 2 days after admission.

## AUTOPSY FINDINGS

Autopsy revealed a well-nourished adult female (BMI: 22.1) with diffuse petechiae of the trunk and focal soft tissue hemorrhage beyond what would be expected for iatrogenic resuscitation efforts, suggesting a coagulopathic process. The joints appeared normal and were without bony deformity or other stigmata of arthritis. There was no palpable lymphadenopathy. The proximal small intestine and duodenum showed mildly hyperemic and granular mucosa. The sigmoid colon was, in contrast, mostly unremarkable with minimal but rather diffuse pale-tan tenacious, adherent mucus. The mesentery was involved by multiple 0.5 – 2.0 cm calcified nodules (thought to be fat necrosis by prior imaging). The adrenal glands were markedly atrophic (2-3 g each [RR: 7-12 g for both]) likely secondary to her prolonged steroid therapy. The liver demonstrated diffuse spongy parenchymal collapse and softening as well as a variegated and congested appearance suggestive of hypovolemic shock. The pericardium was diffusely adherent to both the heart and overlying mediastinal tissues and without effusion. The heart contained numerous yellow vegetations of the cardiac valves, predominantly on the pulmonic and mitral valves. Notably, there were severe adhesions involving part of the bilateral pleura, intestine, and spleen. The bilateral kidneys had coarse cortical pitting (presumably from chronic hypertension) and a hemorrhagic-appearing renal collecting system, possibly due to coagulopathy and complications of shock. The uterus and bilateral ovaries were surgically absent, but no pelvic adhesions were observed. No significant internal hemorrhage or other trauma was identified to help explain the hypovolemic shock.

**Figure 1:**
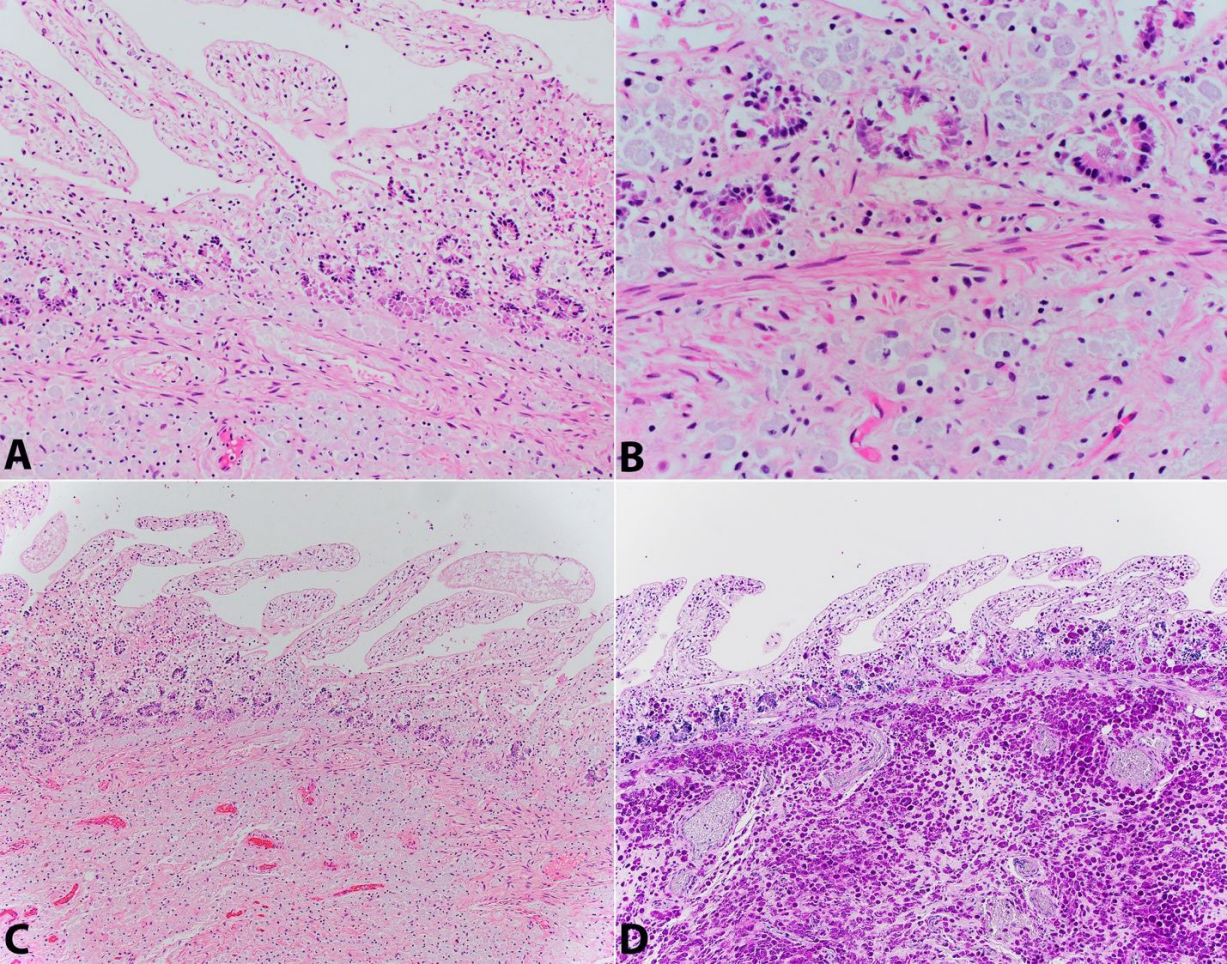
Photomicrographs of the small bowel. **A –** Foamy histiocytes with ingested *T. whipplei* organisms (H&E, 200x); **B –** Mucosa/submucosa transition with foamy histiocytes with ingested *T. whipplei* organisms (H&E, 400x); **C –** Foamy histiocytes with ingested *T. whipplei* organisms (H&E, 100x); **D –** *T. whipplei* organisms positive with PAS-D stain (100x).

**Figure 2:**
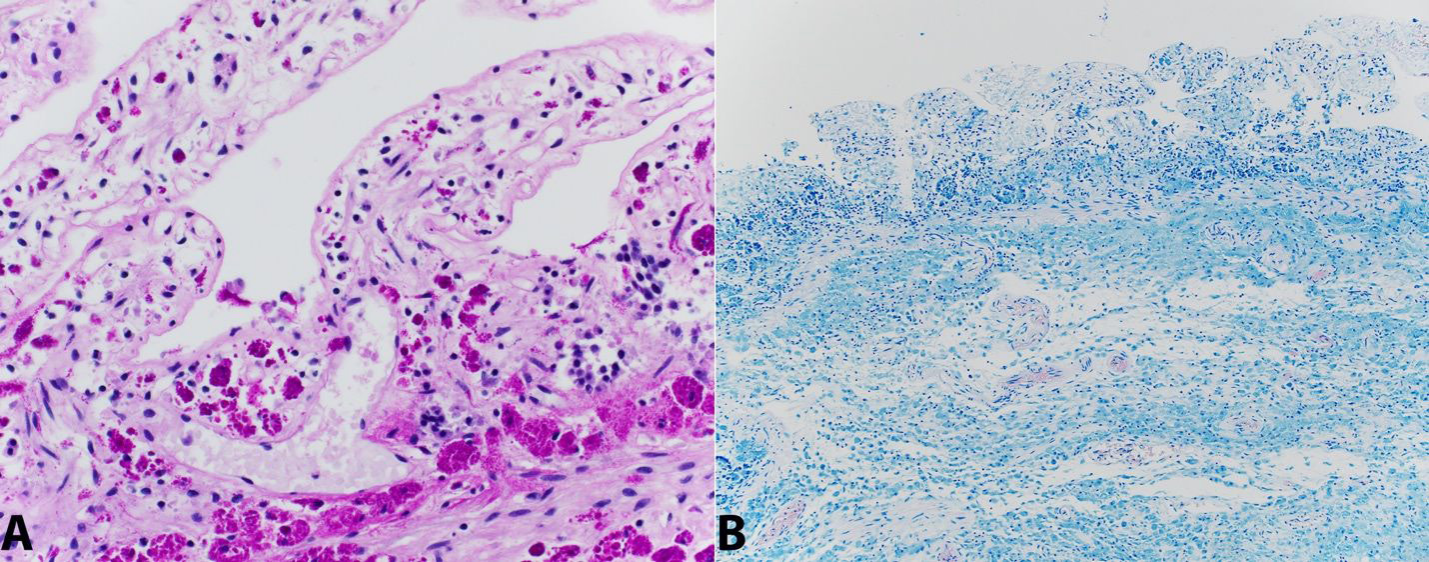
Photomicrograph of the small bowel. **A –** positive PAS-D stain highlighting individual organisms at high power (400x); **B –** Absence of acid-fast bacilli (AFB) on modified Ziehl-Neelsen AFB stain (100x)

**Figure 3:**
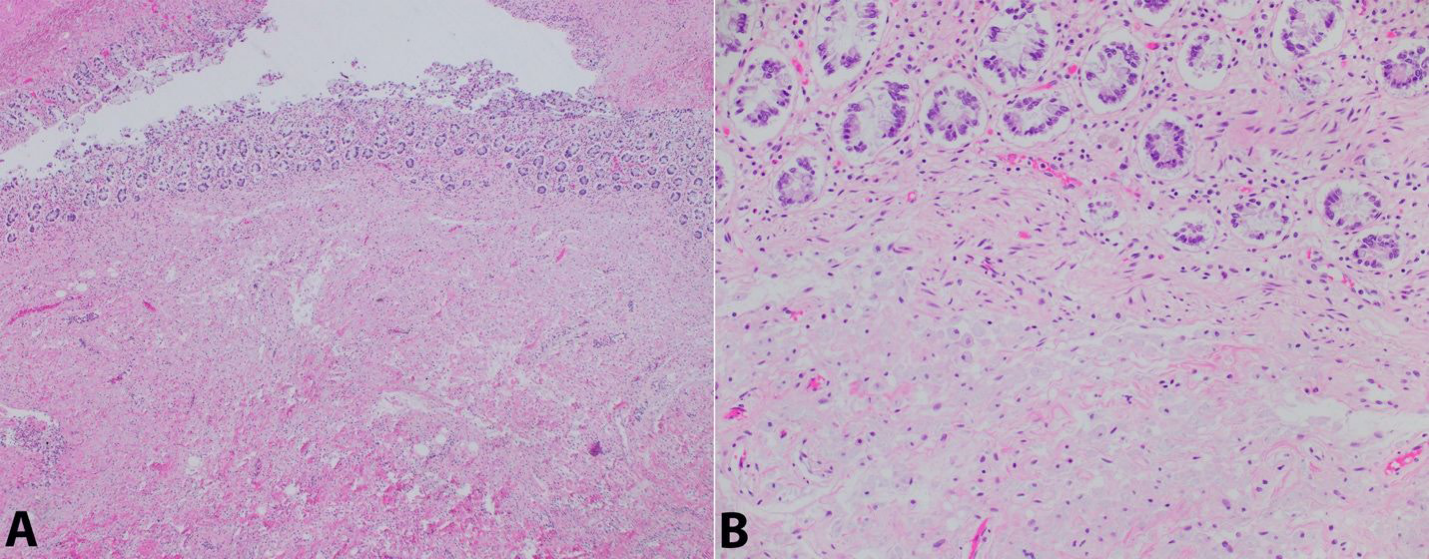
Photomicrograph of the colon. **A –** Right colon demonstrating predominantly lamina propria and mural involvement by foamy histiocytes (H&E, 40x); **B –** Right colon demonstrating predominantly mural involvement by foamy histiocytes (H&E, 200x).

**Figure 4:**
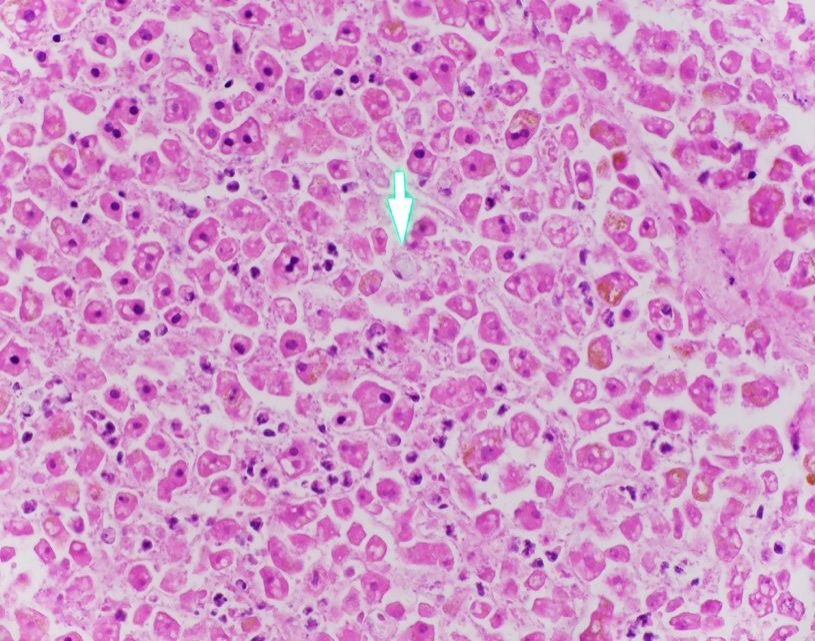
Photomicrograph of the liver with rare foamy histiocyte (arrow) in sinusoid (H&E, 400x).

**Figure 5:**
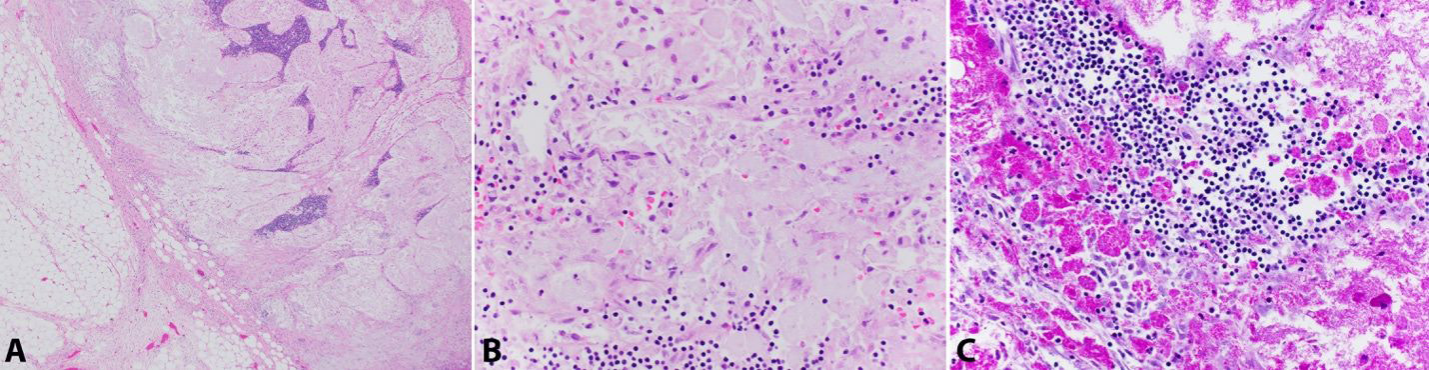
Photomicrograph of the lymph node. **A –** Lymph node edge with sparse lymphoid tissue predominantly replaced by histiocytes (H&E, 40x); **B –** Lymph node with organism laden histiocytes (H&E, 400x); **C** – Lymph node with organisms highlighted by positive PAS-D stain (400x).

**Figure 6:**
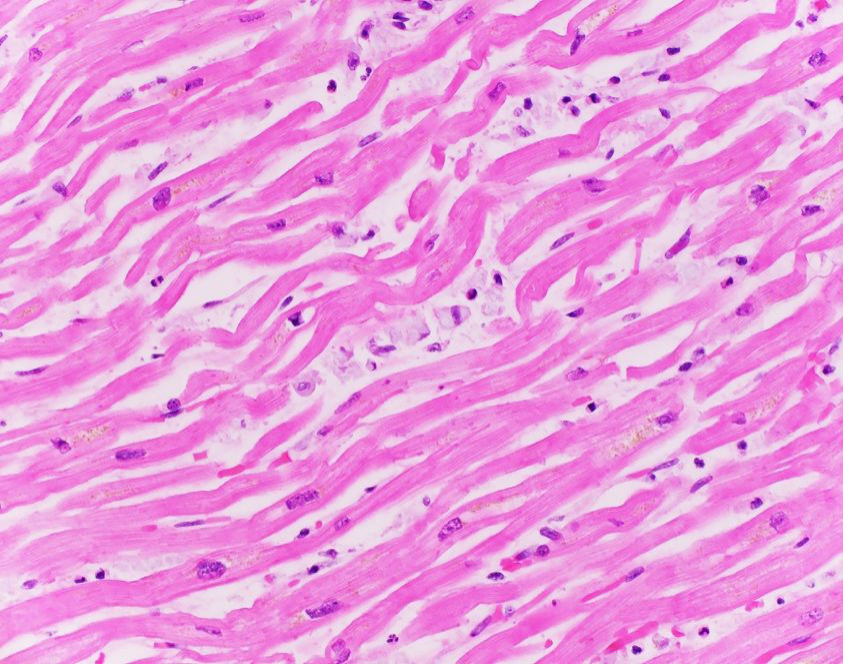
Photomicrograph of the myocardium with rare foamy histiocytes without associated myocarditis (H&E, 400x)

**Figure 7:**
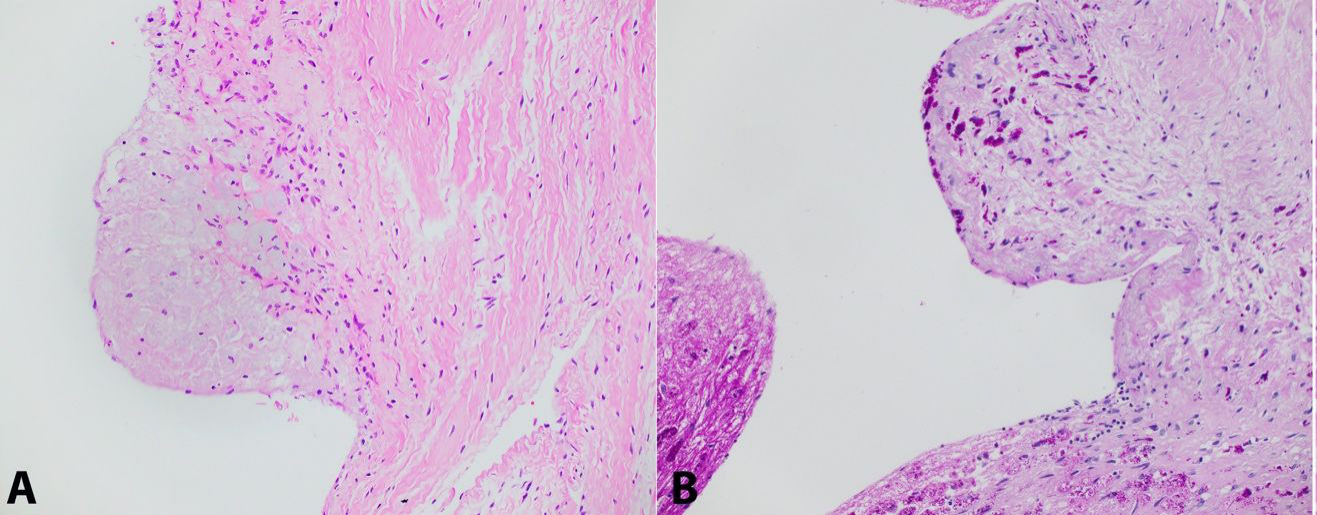
Photomicrograph of the cardiac valves. **A –** Mitral valve vegetation composed of organism laden histiocytes (H&E, 200x); **B –** Mitral valve vegetation, positive PAS-D stain (200x).

Microscopically, widely disseminated bacilliform organisms were identified within the cytoplasm of histiocytes in numerous organs: small and large bowel, mesenteric lymph nodes, pericardium, myocardium, cardiac valves, liver, spleen, and pleura (Figures 1 to 7). The tubular GI tract was the most heavily involved organ system, with organism-laden macrophages minimally involving the lamina propria of the (autolyzed) villi of the small bowel, but more strikingly and densely involving the submucosa with extension into the muscularis propria (Figures 1, 2 and 3). The extent and depth of involvement of the muscularis propria of the GI tract was proximally favored – with significantly decreased disease burden in the left colon and rectum.

At low power the liver appeared uninvolved, but upon close inspection at higher power (400x) rare single blue-tinged macrophages were present in a distribution completely limited to the sinusoids (Figure 4).

The lymph nodes lacked true, well-formed granulomas and were rather diffusely replaced by organism-laden histiocytes with associated calcifications (Figure 5).

The myocardium was sparsely involved with rare macrophages percolating between myocytes, predominantly in an epicardial distribution that was more or less contiguous with the overlying dense involvement along the adherent pericardium (Figure 6).

The involved cardiac valves demonstrated discrete and sharply demarcated aggregates of histiocytes (laden with organisms by PAS-D stain) in an immediately subendocardial distribution that formed plaque-like nodules (Figure 7). There was also an organizing fibrotic tissue response at the periphery of the cardiac vegetations resulting in valvular degeneration.

Splenic involvement was complicated by marked post-mortem autolysis and hemorrhage but could be appreciated particularly in the soft tissues surrounding the hilum.

The intracellular bacilliform organisms in the involved organs were highlighted with PAS-D and later confirmed to be *T. whipplei* by PCR at ARUP laboratories (Figures 1, 2, 5 and 7). Notably, acid fast stains performed on sections of the cardiac valves, lymph nodes, and small bowel were negative, ruling out infection with mycobacterial species (Figure 2B). A tissue Gram stain on the cardiac valves did not adequately visualize the organisms. Collectively, these findings are strong evidence for disseminated, multi-organ involvement with *T. whipplei*. Notably, in the intestine, there was also concurrent focal mild active enteritis also potentially induced by the infection, which may have further contributed to the patient’s severe diarrhea along with the malabsorptve effects of the *T. whipplei* organisms.

Additional microscopic findings included adrenal glands uninvolved by *T. whipplei* organisms, but atrophic appearing, likely due to chronic exogenous steroids. Lastly, no CNS involvement by *T. whipplei* was identified; however, there were focal areas of leptomeningeal fibrosis, which may have been caused by a prior resolved pathologic process such as meningitis, vasculitis, or focal microhemorrhage.

## DISCUSSION

This case of *T. whipplei* mimicking seronegative arthritis in a female patient culminated in hypovolemic shock due to complications of overwhelming *T. whipplei* infection in the setting of severe immunosuppression. The greatest challenge in diagnosing rare infections such as WD is in considering them for inclusion in the differential diagnosis. As this case illustrates, the diverse manifestations of WD and the uncommon presentation in a middle-aged female patient challenged efforts at diagnosis. To our knowledge, this case represents the first or one of the first reported cases of fatal WD developing in the setting of iatrogenic immunosuppression for a diagnosis of seronegative rheumatoid arthritis (RA).

WD is a diagnosis of exclusion that can be considered after excluding the other more common disorders associated with symptoms of fever, arthralgias, GI and neurological symptoms.^9^ Interestingly, while classically described as disease of the GI tract, the reported presentation with GI symptoms varies greatly between studies (~80-95%), and other findings (particularly arthropathy, reportedly present in ~20-85%) can precede development of GI symptoms and may be the primary complaint of the patient.^2^ ^,^ ^5^ ^,^ ^7^ ^,^ ^9^ ^,^ ^14^ ^,^ ^15^ In this case the patient was clinically diagnosed with seronegative rheumatoid arthritis (relatively refractory to nearly 7 years of immunosuppressive treatment) given the constellation of joint symptoms, although no definitive laboratory and imaging findings to fully support the diagnosis. Further clouding the clinical picture, the patient’s clinical symptoms were reported in medical record notes to have improved after initiation of some RA drugs. We speculate that this improvement may have been secondary to a generalized decrease in systemic inflammation; however, this improvement likely mislead clinicians to believe that her presumptive RA was responding to therapy.

The broad spectrum of symptoms associated with WD including diarrhea, arthralgias, and central nervous system (CNS) symptoms raises a differential diagnosis that may include inflammatory bowel disease, connective tissue disease, and other, more common infections such as tuberculosis, AIDS, and chronic diarrheal organisms.^3^ ^,^ ^9^ ^,^ ^16^ In this particular case, the patient’s symptoms of arthralgias, diarrhea, fatigue, and non-specific neurologic symptoms, are likely all attributed to disseminated multi-organ infection by *T. whipplei*.

Several of our autopsy findings help explain the extent of our patient’s involvement by WD and the pathogenesis of her hypovolemic shock. The chronic diarrhea is most likely due to chronic malabsorption, and protein-losing enteropathy from small bowel *T. whipplei* infection. The active enteritis observed microscopically is not a common finding reported in WD, but in our case may be a manifestation of the severity of disease. These GI findings in total not only explain the cause of diarrhea but may also have further contributed to hypovolemic shock, due to the massive fluid and associated protein loss. Further exacerbating the hypovolemic shock, the valvular vegetations, and severe pericardial and pleural adhesions resulting in a restrictive pleuropericarditis that most likely led to further decreased cardiac output and overall cardio-pulmonary dysfunction during the increased demand of cardiac output due to hypovolemia. The pathologic findings of the liver parenchyma included diffuse centrilobular necrosis, which is consistent with the patient’s hypovolemic shock.

*T. whipplei* cannot be detected with traditional blood cultures if there is suspicion of septic shock, which explains why no infectious etiology was clinically detected in this case – and the ultimate reason for the request for autopsy. Laboratory culture for confirmation of WD is both technically challenging and not clinically useful due to the exceptionally slow growth dynamics of *T. whipplei* (replication time estimated at 18 days) and an inability of the organisms to synthesize certain amino acids that are usually supplemented by their environment/host.^9^ ^,^ ^16^ ^,^ ^17^ Non-invasive detection of the organisms in saliva or feces is also not sufficient for the diagnosis of WD due to the relatively high carrier rate in asymptomatic individuals (up to 10% in some studies).^4^ ^,^ ^18^

The autopsy findings also highlight a particular challenge in diagnosing WD by superficial luminal endoscopic biopsy. The bacteria-laden macrophage infiltrates may lie deep to the mucosa (potentially even with transmural muscle involvement), and could potentially go unsampled in superficial biopsies, even in cases with a heavy burden of disease. The distribution of organisms in the small bowel, even in this patient with widely disseminated disease, is most dense in the muscularis propria, which is not typically intentionally sampled on routine biopsies. While admittedly somewhat obscured by the degree of autolysis secondary to autopsy, the number of organisms present in the tips and lacteal of the small bowel villi is relatively sparse. Thus, even a thorough pathologist actively looking for evidence of WD, may not be provided with a sufficient and deep enough sample to make the diagnosis.

The remainder of the autopsy findings support WD as the etiology of this patient’s constellation of long-term signs and symptoms. No vasculitis, mononuclear cell infiltrates, or other auto-immune processes were identified histologically; admittedly, their identification may have been obscured by the degree of disseminated disease in this case. In either scenario, the presence of these findings would not alter the cause of death nor would their absence rule out these diseases. In short, there is no evidence for rheumatologic disease, which also would not explain the diverse symptomatology and severity of this patient’s disease course.

WD disease misdiagnosed as inflammatory rheumatoid disease has been reported in numerous studies, but none such cases appear to have led to a fatal outcome. In a retrospective European cohort of 113 patients diagnosed with WD,^2^ the predominant symptom of all patients was arthralgia (although other cohorts have also suggested arthralgia are not as common of a symptom),^5^ ^,^ ^15^ and roughly half of the cohort was first misdiagnosed with inflammatory rheumatoid disease. Half of these patients were treated with DMARDs, which often resulted in more rapid clinical progression of WD before diagnosis. From the patients who reported arthralgia in this study, two were women but no weight loss or diarrhea was reported. Similar findings of patients with WD presenting rheumatoid-like disease without GI symptoms were found in another large cohort (Glaser 2017).^14^ Yet, in several other studies in undiagnosed WD patients with arthralgia, immunosuppressive therapy resulted in progressive GI symptoms and diarrhea.^19^ ^-^ ^21^ Surprisingly, rare reported cases of WD diarrhea culminating in hypovolemic shock *did not involve* suspected rheumatologic etiologies nor immunosuppressive therapies at all.^22^ ^,^ ^23^ Several of these studies highlight that WD may be commonly misdiagnosed as inflammatory rheumatoid disease and that WD symptoms may be exacerbated with immunosuppressive therapies. This then leaves in question how many similar WD cases are actually fatal and never received a diagnostic post-mortem examination.

Clearly there is no specific symptomatology for WD and its clinical overlap with inflammatory rheumatoid disease poses a challenge for a timely diagnosis by our clinical colleagues. Speculatively, this could be due to patient-specific immune interactions with bacterial antigens, or simply due a chronic inflammatory state and immune dysregulation resulting from the long-term dissemination of a slow-growing pathogen, such as in some disseminated mycobacterial infections. Several research findings support an immunogenetic predisposition to full-fledged manifestation of WD: namely, colonization by *T. whipplei* does not lead to disease in all individuals,^4^ ^,^ ^18^ specific HLA alleles and cytokine gene polymorphisms predispose some individuals to WD development,^24^ ^,^ ^25^ and that patients with WD have reduced titers of antibodies compared to healthy, colonized individuals in addition to other cellular immune dysregulation.^4^ ^,^ ^9^ ^,^ ^18^ ^,^ ^26^ ^-^ ^28^ The heterogeneous and complex interactions of *T. whipplei* with the human immune system is beyond the reach of our current understanding, but may account for the diversity of disease presentation.

This case report highlights the challenge of diagnosing a rare disease with an atypical clinical presentation, but may help serve as a reminder to consider unusual infections in patients with a similar constellation of symptoms. It also underscores the relevance and importance of a thorough autopsy examination, even with today’s complex imaging and laboratory testing. We also would like to highlight the potential sampling discrepancies that may arise with superficial endoscopic biopsies given the distribution of disease (even in cases with a high infectious burden such as this). WD, although while rare, should be considered whenever there are unexplained GI symptoms or arthropathies, with or without cardiac/CNS symptoms, to help gather more accurate and current data about its prevalence in various populations.
